# Secondary Bacterial Infections in Patients With Viral Pneumonia

**DOI:** 10.3389/fmed.2020.00420

**Published:** 2020-08-05

**Authors:** Prasanth Manohar, Belinda Loh, Ramesh Nachimuthu, Xiaoting Hua, Susan C. Welburn, Sebastian Leptihn

**Affiliations:** ^1^Zhejiang University-University of Edinburgh (ZJU-UoE) Institute, Zhejiang University, Haining, China; ^2^School of Medicine, The Second Affiliated Hospital Zhejiang University (SAHZU), Hangzhou, China; ^3^Antibiotic Resistance and Phage Therapy Laboratory, School of Bio Sciences and Technology, Vellore Institute of Technology (VIT), Vellore, India; ^4^Department of Infectious Diseases, Sir Run Run Shaw Hospital, Zhejiang University School of Medicine, Hangzhou, China; ^5^Key Laboratory of Microbial Technology and Bioinformatics of Zhejiang Province, Hangzhou, China; ^6^Infection Medicine, Biomedical Sciences, Edinburgh Medical School, College of Medicine and Veterinary Medicine, The University of Edinburgh, Edinburgh, United Kingdom

**Keywords:** secondary bacterial infection, pulmonary viruses, viral pneumonia, SARS-CoV-2, COVID-19, influenza, SARS, antibiotic resistance

## Abstract

Pulmonary diseases of viral origin are often followed by the manifestation of secondary infections, leading to further clinical complications and negative disease outcomes. Thus, research on secondary infections is essential. Here, we review clinical data of secondary bacterial infections developed after the onset of pulmonary viral infections. We review the most recent clinical data and current knowledge of secondary bacterial infections and their treatment in SARS-CoV-2 positive patients; case reports from SARS-CoV, MERS-CoV, SARS-CoV2 and the best-studied respiratory virus, influenza, are described. We outline treatments used or prophylactic measures employed for secondary bacterial infections. This evaluation includes recent clinical reports of pulmonary viral infections, including those by COVID-19, that reference secondary infections. Where data was provided for COVID-19 patients, a mortality rate of 15.2% due to secondary bacterial infections was observed for patients with pneumonia (41 of 268). Most clinicians treated patients with SARS-CoV-2 infections with prophylactic antibiotics (63.7%, *n* = 1,901), compared to 73.5% (*n* = 3,072) in all clinical reports of viral pneumonia included in this review. For all cases of viral pneumonia, a mortality rate of 10.9% due to secondary infections was observed (53 of 482). Most commonly, quinolones, cephalosporins and macrolides were administered, but also the glycopeptide vancomycin. Several bacterial pathogens appear to be prevalent as causative agents of secondary infections, including antibiotic-resistant strains of *Staphylococcus aureus* and *Klebsiella pneumoniae*.

## Introduction

**Figure 1:**
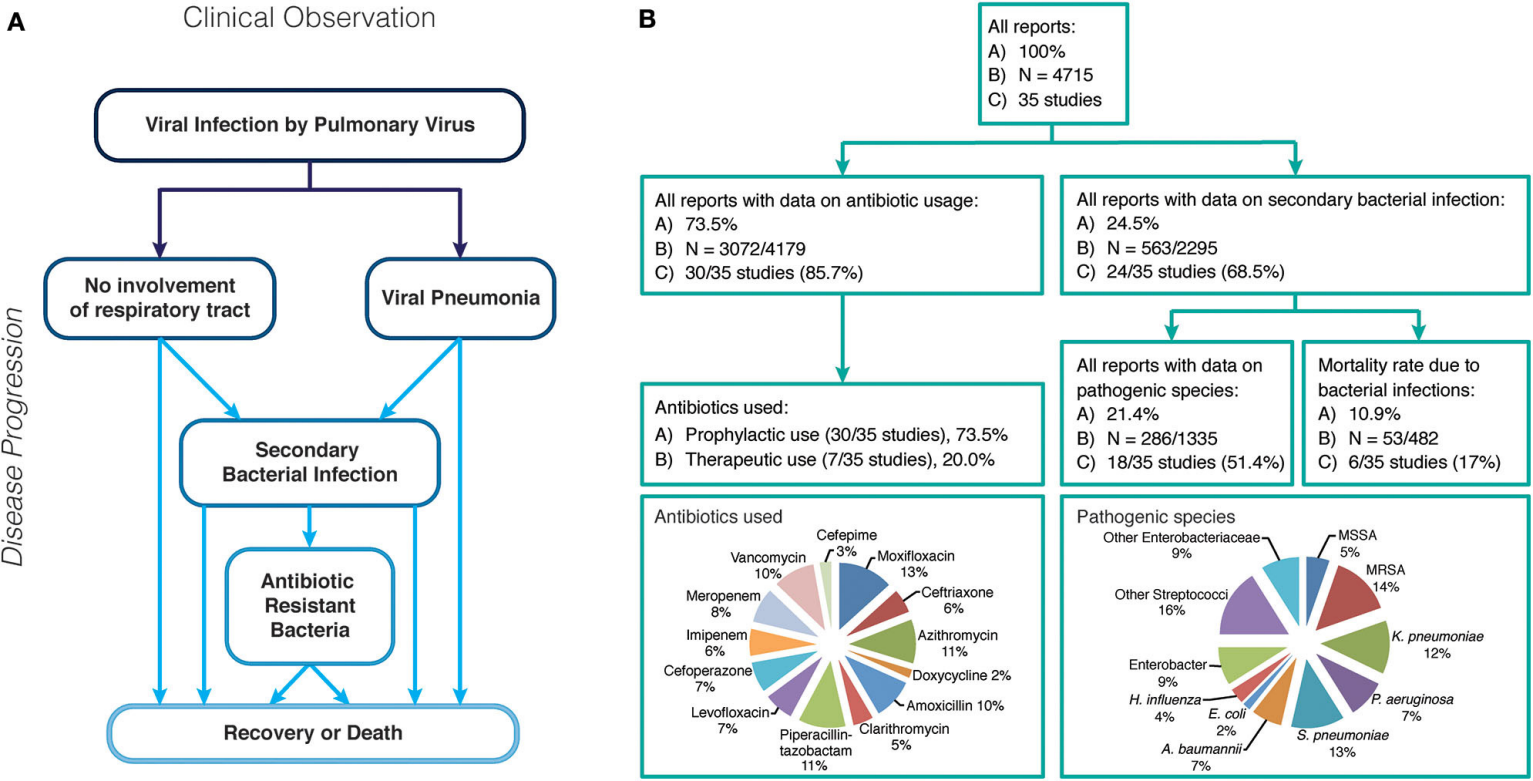
**(A)** Possible disease progression after infection with a respiratory virus. **(B)** Overview of case reports of all respiratory virus infection data reviewed in this study, including rate of secondary bacterial infections, antibiotic use and pathogenic bacteria identified.

Viruses causing respiratory tract infections have been the cause of high morbidities and mortality rates worldwide, often in a seasonal manner for decades (1). In the last 20 years, the world has experienced six major outbreaks of infectious agents (SARS-CoV: 2002-2004; H1N1 Influenza: 2009–2010; MERS-CoV: 2012–2020; Ebola virus: 2013–2016; Zika virus: 2015–2016; SARS-CoV-2: 2019-present), infection with four of which (SARS-CoV, H1N1 Influenza, MERS-CoV, SARS-CoV-2) result in respiratory tract infections. During the emergency of a viral disease, attention is initially focussed on clinical management of the primary infection, but it is imperative to consider secondary bacterial infections that develop in patients during or following initial infection. In addition, co-infections - the simultaneous infection with a second pathogen of viral or bacterial origin- can also occur and ultimately result in the same outcome: The patient suffers from complications caused by two different pathogens (2, 3). One major complication of viral infections, especially pulmonary viruses, is colonization of the viral affected organs by bacteria which is associated with high morbidity and mortality rates (Figure 1A), following a weakened immune response and/or opportunistic and accessible routes of entry for bacterial pathogen(s) (4). While secondary bacterial infections or superinfections are largely a consequence of immune susceptibility caused by the primary viral infection, co-infections are multiple infections (viral/bacterial/yeast) that occur simultaneously. Co-infections, secondary infections or “superinfections” are common during viral pandemics. The 1918 Spanish Flu pandemic saw around 50 million deaths ascribed to bacterial co-infections and during the 2009 H1N1 Influenza pandemic up to 34% of all deaths were a result of bacterial co-infections (5). These complications are often neglected in the clinical record (Figure 1B) (6, 7).

While the precise mechanism for susceptibility to secondary infections is poorly understood, it is likely that virus-mediated immunosuppression of the host innate immune enables opportunistic bacteria to colonize the host as it was shown for *Streptococcus* (8). Most studies on host-pathogen interactions focus on a single-pathogen, one-host model although mammalian hosts are simultaneously exposed to multiple (opportunistic) pathogens (9). The immune response to one pathogen may alter the immunity to other infectious agent(s), resulting in increased prevalence of viral-induced secondary bacterial infections (10). Mammalian cells are more susceptible to bacterial attachment and colonization when infected by a virus (11, 12); infection with respiratory syncytial virus (RSV), rhinovirus and influenza virus (all of which damage the mucosal layer), lead to bacterial adherence of *Streptococcus pneumoniae, Pseudomonas aeruginosa*, and *Haemophilus influenzae*, and biofilm formation on the linings of the airways (13).

Common bacteria isolated during secondary infections include *Staphylococcus aureus, S. pneumoniae, Neisseria meningitides, H. influenzae, Klebsiella pneumoniae* and members of the *Proteus, Enterobacter* and *Citrobacter* spp. (14). During active viral infections, the route of infection, proximity of infection as well as virulence factors varies between bacterial strains resulting in different outcomes. Effective treatment of both the viral and the secondary bacterial infection(s) is of critical importance. Antiviral treatments deployed initially do not treat secondary bacterial infections (15). The preferred treatment for bacterial infections is generally broad-spectrum antibiotics, but this can result in undesirable side effects that have a negative impact on the normal microflora of the host (16–18). Susceptible individuals within a population can be protected from risk of some common bacterial pathogens that are also capable to cause secondary infections, for example pneumonia, if vaccines are available; furthermore, the immune system is often capable of fighting bacterial infections alone, while during a virus-bacterial infection, the complex immune responses might be inapt to eliminate bacterial-induced disease complications (19). To reduce severe disease progression or lethal outcomes, alternative ways to alleviate complications due to secondary bacterial infections and to eliminate bacterial pathogens while preserving host immunity might be necessary.

Secondary infections can also be acquired from the patient's environment i.e., hospital-acquired or nosocomial infections. Nosocomial pathogens are often resistant to a wide range of antibiotics; a result of increased use of antibiotics and decades of over- and misuse resulting in selection for multi-drug resistant pathogens (MDR pathogens) (20). MDR is a global problem with >50,000 people per year infected, of which in ~25% of the cases no effective antibiotic is available (21) as many major pharmaceutical stakeholders have discontinued their search for new chemical antimicrobials (22). Antibiotic resistant organisms include Methicillin-resistant *S. aureus* (MRSA), Drug-resistant *Streptococcus*, Vancomycin-resistant *Enterococci* (VRE), drug-resistant *Mycobacterium*, Carbapenem-resistant *Enterobacteriaceae* (CRE), Colistin-resistant *Klebsiella*, Carbapenem-resistant *Pseudomonas aeruginosa*, and Carbapenem-resistant *Acinetobacter baumannii* (20). If a patient with a viral infection is unfortunate to become infected by MDR bacteria, no standard treatment is currently available. In one COVID-19 cohort study, described in more detail below; almost 50% (10 of 21) patients developed secondary bacterial infections in the lung caused by *Klebsiella pneumoniae, Staphylococcus, Acinetobacter baumannii*, and *E. coli*, ultimately leading to their death, despite receiving prophylactic antibiotic therapy (23).

This evaluation of all the COVID-19 reports available that reference secondary infections showed that most clinicians prescribed meropenem and linezolid to children with a rate of prophylactic treatment between 19.4 and 100.0% (24). In adults mainly quinolones, cephalosporins and macrolides were used for treatment with rates of prophylaxis between 13.2 and 100.0%. In most cases, etiological evidence was not used for therapeutic use of antibiotics.

In this study, we have assimilated the most recent clinical data on bacterial secondary infections associated with pneumonias, caused by pulmonary viruses, including for SARS-CoV-2. We outline treatments used or prophylactic measures employed for secondary bacterial infections. This review focuses on secondary bacterial infections that occur in the respiratory tissue of patients suffering from a viral infection, as most lung infections by secondary bacterial pathogens remain localized in the respiratory tract, while a small percentage leads to infections that spread systematically.

## COVID-19 Related Secondary Bacterial Infections and Treatment

SARS-CoV-2 is an enveloped, positive-sense, single-stranded RNA virus, belonging to the family Coronaviridae. The virus emerged in 2019 and is responsible for respiratory illnesses Coronavirus Disease 2019 (COVID-19) that results in respiratory tract infections and causes severe lung damage in most fatal cases. SARS-CoV-2 has overwhelmed health systems, with numbers of those infected with COVID-19 requiring hospitalization and critical care.

Early stages of SARS-CoV-2 infection typically include symptoms of mild fever and shortness of breath (25). As the virus replicates in the respiratory tract, the symptoms worsen and once the virus reaches and begins infecting the alveoli, the host responds by generating inflammatory factors in alveolar tissues that may result in pneumonia. In almost all severe cases, SARS-CoV-2 infection results in pneumonia and the inflamed fluid-filled alveolar tissue now is an ideal habitat for bacterial growth for pathogens including *P. aeruginosa* and *S. aureus*. The causative agent resulting in a severe disease progression may be bacteria, virus or fungi (26).

The presence of secondary bacterial infections in those infected with SARS-CoV-2 complicates treatment (26, 27). An overview of all COVID-19 case studies, available as Supplementary Information, where we have analyzed 15 case studies reporting SARS-CoV-2 infection (Supplemental Data Sheet 1). In particular for data obtained from clinical observations in China, COVID-19 patients are most commonly treated with antibiotics (azithromycin, moxifloxacin, ceftriaxone, vancomycin, cefepime) to reduce the risk of nosocomial infections, a prophylactic strategy. Should a bacterial infection occur despite the prophylactic use of antibiotics, caused by bacteria that are resistant to one or more drugs, different combinations of the same drugs are being used for the treatment, as a therapeutic strategy. Both strategies, the prophylactic and the therapeutic use of antibiotics, deploy the same types and dosage of drugs: however the prophylactic use of antibiotics is not recommended by most health institutions and governments around the globe, due to the observed increase in antibiotic resistance rates, which correlate with the overuse (and misuse) of antibiotics (28, 29). However, the risk of superinfection with MDR bacteria poses additional challenges for the treatment of severely sick COVID-19 patients in intensive care units (ICU).

## Case Reports of Antibacterial Therapy in COVID-19 Patients

Fifteen clinical reports, for which data for secondary infections were available, mainly from hospitals in China, were examined to provide information of the occurrence, treatment of or prophylactic measures for bacterial secondary infections during the course of COVID-19 infections (Supplementary File). A total of 3,133 COVID-19 patient cases were detailed in the 15 reports (25–39). Prophylactic antibiotic therapy was given to 1,901 of the 2,983 patients (63.7%). We excluded one study (patient *n* = 150), as they did not report details on the antibiotic therapy (31) and 5/14 studies did not report the type of antibiotics used. The most commonly used antibiotics were moxifloxacin (*n* = 452, 39.2%) and azithromycin (*n* = 294, 30.7%). Others include ceftriaxone, vancomycin, cefepime, cephalosporins, quinolones, carbapenems, tigecycline, doxycycline, and cefoperazone. In total, 10 studies reported secondary bacterial infections, with 273 of the 997 patients (27.3%) being infected. Again, we excluded 5 studies (patients *n* = 2,136), which did not report any information on any clinical complications other than caused by the SARS-CoV-2 virus (28, 30, 34, 35, 37). The most common bacterial infections were caused by the Gram-positive pathogen *Staphylococcus aureus* or by strains of the Gram-negative bacterium *Klebsiella pneumoniae*. Five studies reported infections that were confirmed by tests in a microbiology laboratory, which identified MRSA, MSSA, *Serratia, Acinetobacter, Pseudomonas, E. coli, H. influenza*, and *E. cloacae* (patients *n* = 16) (25, 26, 29, 36, 38). Three studies reported therapeutic antibiotic treatment in 92 of 146 patients (63%), which were administered as secondary bacterial infections were observed (29, 35, 39). Four studies reported complications with fatal outcome due to bacterial infections that included 41 of 268 patients (15.2%) (26, 27, 33, 39). An overview of the studies can be found in Supplementary Figure 1, which also provides the information on percentages of antibiotic use and types of antibiotic compounds, among others.

These clinical reports provide insights into the complications and challenges faced in treating SARS-CoV-2 infected patients. Patients with pneumonia caused by other respiratory viruses or SARS-CoV-2 pneumonia patients were often prophylactically treated with broad-spectrum antibiotics, in an attempt to reduce the risk of bacterial superinfections. Complications were observed in patients that were not treated with antibiotics. Antibiotic-resistant infections, in particular, nosocomial - hospital-acquired - infections were common for more than one third of ICU patients, posing a threat to disease progression, often resulting in the death of the affected individual (26, 40).

## SARS-related Secondary Bacterial Infections and Treatment

Severe Acute Respiratory Syndrome-related Coronavirus (SARS) Co-V, was first report in Guangdong Province, China in November 2002. At its initial outbreak (until August 2003), 8,096 probable cases were reported with an ~10% fatality rate (41). Further sporadic cases were reported in November 2003 through April 2004 (42). The case studies showed the occurrence of bacterial co-infections in the large number of patients. These include infections by MRSA, *Klebsiella* spp., *P. aeruginosa* and *Streptococcus* (43). The large multinucleated cells observed in the lungs of the SARS patients may be due to the underlining bacterial infections (43). The use of broad-spectrum antibiotics to cover the secondary bacterial infections is one of the supportive cares during SARS outbreak (44) (Supplementary File). The case studies (*n* = 7) reported the disease progression of a total of 534 SARS-CoV patients (45–51) (Supplementary File). Details about prophylactic antibiotic therapy was reported in five studies (patients *n* = 385) and 100% patients were treated with prophylactic antibiotic therapy (48, 50–53). The antibiotics used for prophylactic treatment were levofloxacin, clarithromycin, amoxicillin-clavulanic acid, piperacillin-tazobactum, maxipime, azithromycin, amoxicillin-clavulanate, cefoperazone/sulbactam, quinolone plus azithromycin, levofloxacin, moxifloxacin, and ceftriaxone plus azithromycin, and most commonly azithromycin (*n* = 206, 74.6%). Of the four studies that reported secondary bacterial infections, 35 of 306 patients (11.4%) became infected. The pathogens that caused the infections included *P. aeruginosa*, MRSA, *S. pneumoniae, K. pneumoniae, Enterococci, Acinetobacter baumannii, Enterobacter, H. influenza, S. maltophilia*, and *Serratia* (45, 47, 49, 51). Despite the deployment of prophylactic antibiotics, the use of therapeutic antibiotics was reported in three studies, administering amoxicillin-clavulanate, clarithromycin, azithromycin, levofloxacin, moxifloxacin and ceftriaxone plus azithromycin (47, 49, 51). In total, 12 patients out of 23 (52%, described in two studies who provided the respective information) had complications due to secondary bacterial infections (47, 51).

During the 2002 outbreak, most SARS patients were treated with broad-spectrum antibiotics as a prophylactic therapy. Contributing factors on antibiotic resistance had not been fully established by the early 2000's; however the use of antibiotics as a precaution in many viral infections and their potential overuse may have contributed to the current antibiotic crisis.

## MERS-related Secondary Bacterial Infections and Treatment

The first cases of the Middle East respiratory syndrome (MERS) CoV epidemic occurred in June 2012 in Saudi Arabia with later outbreaks observed in 2015 and 2018. To date around 2,519 cases have been reported from more than 20 countries with a high fatality rate 34.3% (*n* = 866), the highest among all coronavirus diseases (52). The infection often starts as a mild viral respiratory illness but can rapidly progress to a fatal viral pneumonia mostly complicated by ARDS (acute respiratory distress syndrome) and ARF (acute renal failure) (53). Due to the high mortality rate of MERS infections, the impact of the occurrence of bacterial infections remains unclear as death occurs due to hypoxemia and bilateral interstitial infiltrates in most cases, or ARDS. Nosocomial bacterial pneumonia is however common among MERS patients with ventilator support. Therefore, antibiotic therapy was commonly deployed with secondary bacterial infections rarely occurring under this treatment regime (53–55). Here, we review MERS-related case studies, outlining antibiotic therapies and bacterial infections. A total of 138 MERS-CoV infected patients were reported (55–59) (Supplementary File). The use of prophylactic antibiotic therapy was reported in 4 out of 5 studies. Sixty eight patients were treated with the antibiotics piperacillin-tazobactam, azithromycin, ceftriaxone and trimethoprim- sulfamethoxazole, levofloxacin, imipenem and other broad-spectrum antibiotics (55, 56, 58, 59). The development of secondary bacterial infection was reported in two studies, with 32 of 82 patients experiencing complications due to bacterial pathogens (55, 57). The most common bacteria were MRSA, while others included carbapenem-resistant *Acinetobacter baumannii*, VRE and *S. pneumoniae*. No detailed report was provided about treatment antibiotics and one study reported (patients *n* = 15) complications due to bacterial infections in intensive care units (55).

## Influenza Viruses and Secondary Bacterial Infections

Influenza viruses are known to cause seasonal flu every year and of the four influenza types, type A and B are known to cause seasonal epidemics (60). The influenza A (H1N1) outbreak in 2009 had even developed into a global pandemic while causing seasonal flu epidemics each year. In spite of the availability of vaccines, the global death during the flu season was estimated to be between 0.25 and 0.5 million people according to the WHO (61). Secondary bacterial infections are one of the leading causes for influenza associated deaths (62). According to the Center for Disease Control and Prevention (CDC), during the 2009 H1N1 influenza pandemic between 29 and 55% of mortalities were due to secondary bacterial infections (63). The lethal synergism between influenza virus and *Pneumococcus* strains accounts for the majority of diseases as well as mortality during influenza epidemics (64). Here, we summarize case studies that are related to influenza viruses and secondary bacterial infections (Supplementary File). Details on treatment and disease progression of a total of 910 patients that were infected with influenza were reported in eight case studies (65–72). Five studies reported the deployment of prophylactic antibiotic therapy in 718 of 743 patients (96.6%) (65–67, 71, 72). The most commonly used antibiotics were vancomycin (*n* = 317, 46.2%) and imipenem/meropenem (*n* = 243, 54.7%). Others were azithromycin, ceftriaxone, cephalosporin, piperacillin-tazobactam, linezolid, vancomycin plus cefepime, vancomycin plus meropenem, clarithromycin, levofloxacin and dicloxacillin. In total, 223 patients of 910 (24.5%) developed secondary bacterial infections. The infective agents were *S. pneumoniae*, MRSA, *P. aeruginosa, S. viridians, S. hominis, Enterococci, H. influenza, S. pyogenes, S. mitis, S. agalactiae, Acinetobacter baumannii, E. coli, Chlamydophila pneumoniae*, and *Moraxella catarrhalis*. To treat the infections, one of the studies reported the use of azithromycin, vancomycin, ceftriaxone and cephalosporin (65). In general, all eight case studies reported the treatment complications due to secondary bacterial infections.

## Discussion

The lack of detailed clinical data regarding secondary bacterial infections following COVID-19, SARS, MERS and influenza is surprising. In addition, precise numbers of clinical cases of COVID-19 or other respiratory virus infections with antibiotic-resistant secondary bacterial infections are not available. As globally mortality rates from antibiotic-resistant bacterial infections are increasing, providing such data of secondary infection is important, almost critical.

Clinical reports are a valuable source for biomedical scientists for the advancement of treatment; however, in many cases crucial data such as medical compounds, their dosage, and duration of the therapy, is often not reported, similar to details on patient data or the species of e.g., a pathogenic bacterium. Such data are essential for the study of secondary infections in human patients; the scientific community and ultimately the patient will benefit when clinicians and non-clinical disease experts work close together. The information from such collaborative studies can provide a guideline for treatment. At the same time, the lack of knowledge and studies that should be done in the future is evident. Reviewing the available information, it becomes clear that secondary bacterial infections play a critical role in the morbidity and mortality rates of patients initially falling ill with pulmonary viral diseases. Similar to epidemics caused by influenza viruses, during the current SARS-CoV-2 pandemic, antibiotic-resistant bacterial infections are a significant threat to the well-being of hospitalized COVID-19 patients. Nosocomial infections including ventilator-associated infections are often unavoidable and especially so during a pandemic. The use of broad-spectrum antibiotics is often a routine preventative measure and until alternative treatments and to this date unavailable preventive measures are in place at hospitals, secondary infections will remain unavoidable due to the nature of nosocomial and opportunistic pathogens in combination with the rise of multidrug resistance.

COVID-19 is likely to reappear in populations until a vaccine is deployed, globally. To prepare for recurrent waves of COVID-19 disease and the inevitable next new pandemic, new antibiotics or alternative treatments targeted against secondary bacterial infections are needed. Alternative therapies such as Antimicrobial Peptides or Phage Therapy are being explored and show promise. Most antimicrobial peptides perturb the structure of the bacterial cell envelope while not affecting the eukaryotic cell membrane; eventually creating a leaky lipid bilayer and the collapse of the proton-motif-force, ultimately resulting in bacterial cell death (73–76). Phage therapy makes use of bacterial viruses (or bacteriophages) that specifically infect bacterial pathogens for viral replication, which leads to the lysis of the host when phage progeny is being released (77–83). Anti-virulence compounds are substances that do not kill or arrest the growth of a bacterial pathogen, but rather target pathogen-intrinsic components important for the infection process. These include bacterial components such as exo- and endotoxins, i.e., actively secreted toxins (e.g., hemolysins) or cell-inherent structures that are toxic to the patient, such as LPS, and other factors which facilitate adhesion, invasion or colonization, but also the escape of the host's immune system. Anti-virulence compounds have been demonstrated to prevent or reduce the severity of symptoms of acute infections (84, 85).

Pulmonary coronaviruses will likely be a clinical challenge for many years to come. Viral pandemics from coronaviruses and emerging pathogens are inevitable in a globalized world with interconnected societies, travel and commerce. We should be well-prepared for the next pandemic, exploring and establishing new avenues to treat bacterial pathogens commonly observed in secondary infections to avert a healthcare crisis due to antibiotic-resistance.

## Author Contributions

PM and SL: initial idea. RN and XH: review and comments. BL and SL: figures. PM, BL, SW, and SL: concept and writing. All authors contributed to the article and approved the submitted version.

## Conflict of Interest

The authors declare that the research was conducted in the absence of any commercial or financial relationships that could be construed as a potential conflict of interest.

## Supplementary Material

Supplementary Figure 1 Overview of case reports of SARS-CoV-2 infection data reviewed in this study, including rate of secondary bacterial infections, antibiotic use and pathogenic bacteria identified.

Supplementary File Tables summarizing case reports of patients with pulmonary virus infections and clinical interventions to prevent or cure secondary bacterial infections.

Supplemental Data Sheet 1 Case reports of patients with secondary bacterial infections.
