# Supplementary figures and images for: Secondary Bacterial Infections in Patients With Viral Pneumonia
Source: Front Med (Lausanne). 2020 Aug 5;7:420. doi: 10.3389/fmed.2020.00420 (PMC7419580; doi:10.3389/fmed.2020.00420)

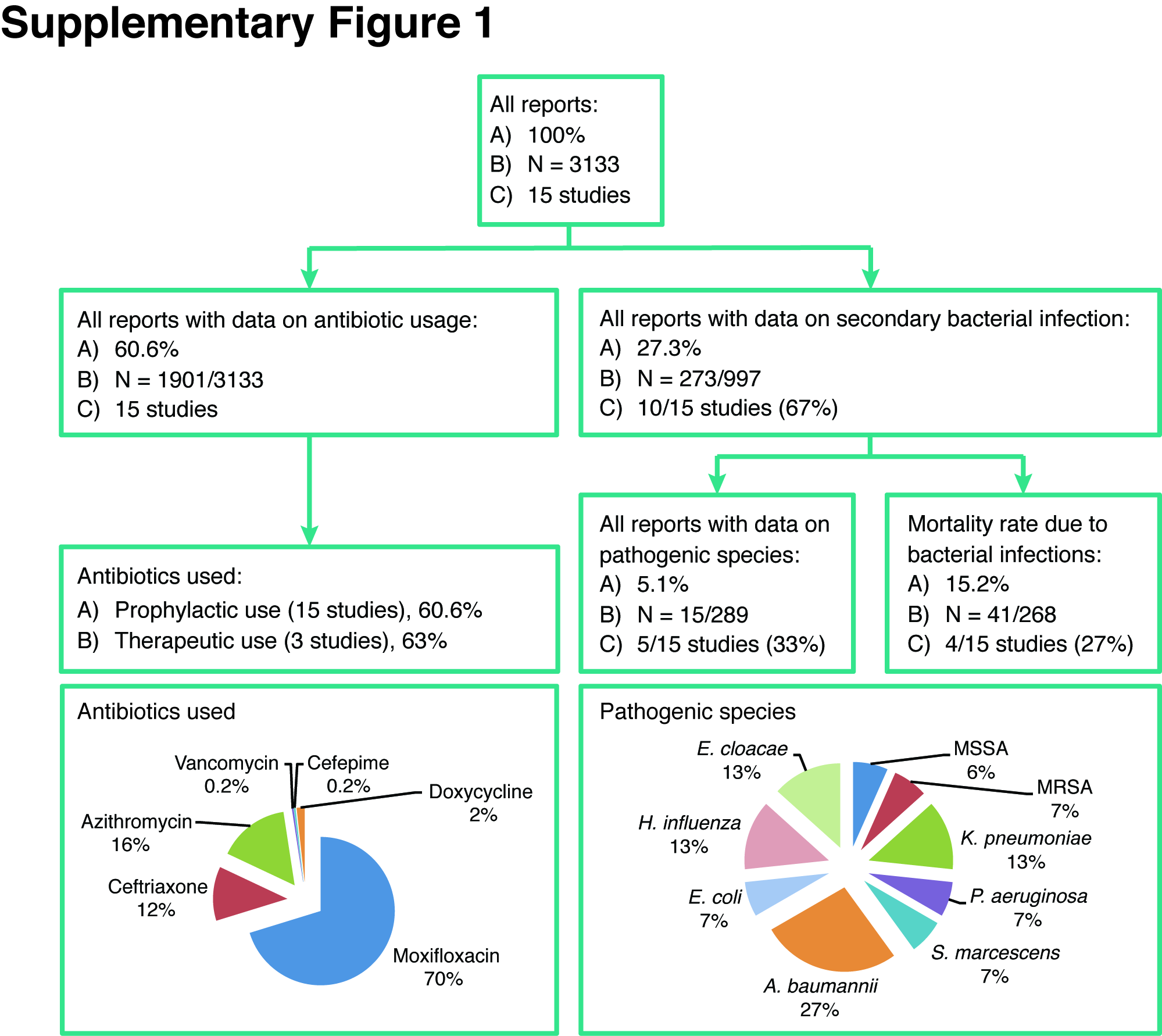

Supplement: Supplementary Figure 1 — Overview of case reports of SARS-CoV-2 infection data reviewed in this study, including rate of secondary bacterial infections, antibiotic use and pathogenic bacteria identified. [file Image_1.TIF]
