# Supplementary material for: Secondary Bacterial Infections in Patients With Viral Pneumonia
Source: Front Med (Lausanne). 2020 Aug 5;7:420. doi: 10.3389/fmed.2020.00420 (PMC7419580; doi:10.3389/fmed.2020.00420)
Supplement: Supplementary File — Tables summarizing case reports of patients with pulmonary virus infections and clinical interventions to prevent or cure secondary bacterial infections. [file Table_1.pdf]

Supplementary Table:

S. Table 1. Case reports of patients with pulmonary virus infections, mainly SARS-CoV-2, and clinical interventions to prevent or cure secondary bacterial infections.

Total Patient number	Development of viral Pneumonia (n)	Development of secondary infection (n)	Prophylactic Antibiotics	Therapeutic Antibiotics	Fatality due to viral pneumonia (n)	Reference
N=138 (SARS-CoV-2)	27 (only ARDS, 19.5%)	N/D	138 (100%) - Moxifloxacin (89, 64.4%), ceftriaxone (34, 24.6%), and azithromycin (25, 18.1%)	N/D	6 (22.2%)	(1)
N=1 (SARS-CoV-2)	0	MRSA	Vancomycin, cefepime	Vancomycin, cefepime	0	(2)
N=1099 (SARS-CoV-2)	1007 (91.6%)	N/D	637 (58%)	N/D	15 (1.4%)	(3)
N=52 (SARS-CoV-2)	52 (100%)	7 (13.5%) - CR *K. pneumoniae* (1), ESBL-positive *K. pneumoniae* (1), ESBL-positive *P. aeruginosa* (1), ESBL-negative *Serratia marcescens* (1) (3 were fungal infections)	49 (94%)	N/D	32 (61.5%)	(4)
N=191 (SARS-CoV-2)	N/D	21 (40%)	181 (95%)	N/D	N/D	(5)

N=150 (SARS-CoV-2)	N/D	16% (11/68 death)	N/D	N/D	N/D	(6)
N=201 (SARS-CoV-2)	201 (100%)	148 (73.6%)	196 (97.5%)	N/D	44 (22%)	(7)
N=99 (SARS-CoV-2)	99 (100%)	1 (1%) - One patient with *A. baumannii* and *K. pneumoniae*	Cephalosporins, quinolones, carbapenems, tigecycline	N/D	11 (11%)	(8)
N=41 (SARS-CoV-2)	41 (100%)	4 (9.7%)	41 (100%)	N/D	6 (15%)	(9)
N=80 (SARS-CoV-2)	55 (68.7%)	N/D	73 (91%), moxifloxacin	N/D	N/D	(10)
N=20 (SARS-CoV-2)	6 (30%)	N/D	Azithromycin (100%)	Azithromycin (100%)	N/D	(11)
N=68 (SARS-CoV-2)	1 (1.4%)	4 (5.8%) – MSSA (n=1), *E. coli* (n=1) and *H. influenza* and *H. influenza* (n=2)	8 (11.7%) – doxycycline (n=5) and moxifloxacin (n=3)	N/D	0	(12)
N=799 (SARS-CoV-2)	N/D	N/D	249 (31.1%) - moxifloxacin, cefoperazone and azithromycin	N/D	N/D	(13)
N=69 (SARS-CoV-2)	N/D	5 (7.2%) - *E. cloacae* (n=2) and *A. baumannii* (n=3)	66 (95.6%) - moxifloxacin (n=38)	N/D	N/D	(14)
N=125 (SARS-CoV-2)	N/D	71 (56.8%)	125 (100%)	71 (56.8%)	N/D	(15)

N/D - No data available; MSSA – Methicillin-sensitive *Staphylococcus aureus*; CR – Carbapenem-resistant; ESBL – Extended-spectrum beta-lactamase;

References:

1. Wang D, Hu B, Hu C, Zhu F, Liu X, Zhang J, Wang B, Xiang H, Cheng Z, Xiong Y, Zhao Y. 2020. Clinical characteristics of 138 hospitalized patients with 2019 novel coronavirus–infected pneumonia in Wuhan, China. Jama 323(11):1061-1069.
2. Holshue ML, DeBolt C, Lindquist S, Lofy KH, Wiesman J, Bruce H, Spitters C, Ericson K, Wilkerson S, Tural A, Diaz G. 2020. First case of 2019 novel coronavirus in the United States. N Engl J Med 382:929-936.
3. Guan WJ, Ni ZY, Hu Y, Liang WH, Ou CQ, He JX, Liu L, Shan H, Lei CL, Hui DS, Du B. 2020. Clinical characteristics of coronavirus disease 2019 in China. N Engl J Med.
4. Yang X, Yu Y, Xu J, Shu H, Liu H, Wu Y, Zhang L, Yu Z, Fang M, Yu T, Wang Y. 2020. Clinical course and outcomes of critically ill patients with SARS-CoV-2 pneumonia in Wuhan, China: a single-centered, retrospective, observational study. The Lancet Respir Med. In-press
5. Zhou F, Yu T, Du R, Fan G, Liu Y, Liu Z, Xiang J, Wang Y, Song B, Gu X, Guan L. 2020. Clinical course and risk factors for mortality of adult inpatients with COVID-19 in Wuhan, China: a retrospective cohort study. The Lancet 395(10229): 1054-1062.
6. Ruan Q, Yang K, Wang W, Jiang L, Song J. 2020. Clinical predictors of mortality due to COVID-19 based on an analysis of data of 150 patients from Wuhan, China. Intensive Care Med 3:1-3.
7. Wu C, Chen X, Cai Y, Zhou X, Xu S, Huang H, Zhang L, Zhou X, Du C, Zhang Y, Song J. 2020. Risk factors associated with acute respiratory distress syndrome and death in patients with coronavirus disease 2019 pneumonia in Wuhan, China. JAMA Intern Med.
8. Chen N, Zhou M, Dong X, Qu J, Gong F, Han Y, Qiu Y, Wang J, Liu Y, Wei Y, Yu T. 2020. Epidemiological and clinical characteristics of 99 cases of 2019 novel coronavirus pneumonia in Wuhan, China: a descriptive study. The Lancet 395(10223):507-13.
9. Huang C, Wang Y, Li X, Ren L, Zhao J, Hu Y, Zhang L, Fan G, Xu J, Gu X, Cheng Z. 2020. Clinical features of patients infected with 2019 novel coronavirus in Wuhan, China. The Lancet 395(10223):497-506.
10. Wu J, Liu J, Zhao X, Liu C, Wang W, Wang D, Xu W, Zhang C, Yu J, Jiang B, Cao H. 2020. Clinical Characteristics of Imported Cases of COVID-19 in Jiangsu Province: A Multicenter Descriptive Study. Clin Infect Dis.
11. Gautret P, Lagier JC, Parola P, Meddeb L, Mailhe M, Doudier B, Courjon J, Giordanengo V, Vieira VE, Dupont HT, Honoré S. 2020. Hydroxychloroquine and azithromycin as a treatment of COVID-19: results of an open-label non-randomized clinical trial. Int J Antimicrob Agents: 105949.
12. Easom N, Moss P, Barlow G, Samson A, Taynton T, Adams K, Ivan M, Burns P, Gajee K, Eastick K, Lillie PJ. 68 Consecutive patients assessed for COVID−19 infection; experience from a UK regional infectious disease unit. Influenza and Other Respiratory Viruses. 2020 Jan 1.
13. Chen T, Wu D, Chen H, Yan W, Yang D, Chen G, Ma K, Xu D, Yu H, Wang H, Wang T. Clinical characteristics of 113 deceased patients with coronavirus disease 2019: retrospective study. Bmj. 2020 Mar 26;368.
14. Wang Z, Yang B, Li Q, Wen L, Zhang R. Clinical features of 69 cases with coronavirus disease 2019 in Wuhan, China. Clinical Infectious Diseases. 2020 Mar 16.
15. Wang R, Pan M, Zhang X, Fan X, Han M, Zhao F, Miao M, Xu J, Guan M, Deng X, Chen X. Epidemiological and clinical features of 125 Hospitalized Patients with COVID-19 in Fuyang, Anhui, China. International Journal of Infectious Diseases. 2020 Apr 11.

S. Table 2. Case reports of patients with pulmonary virus infections, mainly SARS-CoV, and clinical interventions to prevent or cure secondary bacterial infections.

Total Patient number	Development of viral Pneumonia (n)	Development of secondary infection (n)	Prophylactic Antibiotics	Therapeutic Antibiotics	Fatality due to viral pneumonia (n)	Reference
N=14 (SARS-CoV)	11 (78.5%)	7 (50%) - *P. aeruginosa*, MRSA, *Streptococcus* spp., *Klebsiella* spp., *Enterococcus* spp., *Acinetobacter baumannii* and *Enterobacter* spp.	N/D	N/D	11 (100%)	(1)
N=31 (SARS-CoV)	0	N/D	Levofloxacin (500 mg/day) or clarithromycin (500 mg twice daily), and amoxicillin and clavulanic acid (375 mg thrice daily). Piperacillin and tazobactum (n=7)	N/D	0	(2)
N=138 (SARS-CoV)	112 (81.1%)	5 (3.6%) - *H. influenza* (3), *S. pneumoniae* (1) and *K. pneumoniae* (1)	N/D	Amoxicillin-clavulanate and clarithromycin	5 (3.6%)	(3)

Total Patient number	Development of viral Pneumonia (n)	Development of secondary infection (n)	Prophylactic Antibiotics	Therapeutic Antibiotics	Fatality due to viral pneumonia (n)	Reference
N=10 (SARS-CoV)	3 (30%)	N/D	Beta-lactam antibiotics (augmentin, rocephin or maxipime) and a macrolide (clarithromycin or azithromycin) twice daily for four days	N/D	2 (20%)	(4)
N=78 (SARS-CoV)	78 (100%)	7 (9%) – 1 methicillin-resistant *S. epidermidis*, 3 *S. maltophilia*, 2 *K. pneumoniae*, 1 *E. coli*	78 (100%) – amoxicillin-clavulanate, azithromycin / levofloxacin	Amoxicillin-clavulanate, azithromycin / levofloxacin	5 (7%)	(5)
N=190 (SARS-CoV)	36 (18.9%)	N/D	40 cases - cefoperazone/ sulbactam (2 g twice daily); 30 cases - fluoroquinolone plus azithromycin (0.4 g/ day); 60 cases - quinolone plus azithromycin (0.4 g/ day); 60 cases - levofloxacin (0.2 g twice daily) plus azithromycin (0.6 g/ day)	N/D	11 (5.6%)	(6)

Total Patient number	Development of viral Pneumonia (n)	Development of secondary infection (n)	Prophylactic Antibiotics	Therapeutic Antibiotics	Fatality due to viral pneumonia (n)	Reference
N=76 (SARS-CoV)	76 (100%)	16 (21%) – 4 MRSA; 2 MRSE; 3 Enterococci; 3 *A. baumannii*; 2 *K. pneumoniae*; 1 *E. cloacae*; 1 *S. marcescense*	Moxifloxacin alone or ceftriaxone plus azithromycin	Moxifloxacin alone or ceftriaxone plus azithromycin	15 (19.7%)	(7)

N/D - No data available; MRSA – Methicillin-resistant *Staphylococcus aureus*; MRSE – Methicillin-resistant *Staphylococcus epidermitis*

References:

1. Chong PY, Chui P, Ling AE, Franks TJ, Tai DY, Leo YS, Kaw GJ, Wansaicheong G, Chan KP, Ean Oon LL, Teo ES. Analysis of deaths during the severe acute respiratory syndrome (SARS) epidemic in Singapore: challenges in determining a SARS diagnosis. Archives of pathology & laboratory medicine. 2004 Feb;128(2):195-204.
2. So LK, Lau AC, Yam LY, Cheung TM, Poon E, Yung RW, Yuen KY. Development of a standard treatment protocol for severe acute respiratory syndrome. The Lancet. 2003 May 10;361(9369):1615-7.
3. Lee N, Hui D, Wu A, Chan P, Cameron P, Joynt GM, Ahuja A, Yung MY, Leung CB, To KF, Lui SF. A major outbreak of severe acute respiratory syndrome in Hong Kong. New England Journal of Medicine. 2003 May 15;348(20): 1986-94.
4. Tsang KW, Ho PL, Ooi GC, Yee WK, Wang T, Chan-Yeung M, Lam WK, Seto WH, Yam LY, Cheung TM, Wong PC. A cluster of cases of severe acute respiratory syndrome in Hong Kong. New England Journal of Medicine. 2003 May 15;348(20):1977-85.
5. Peiris JS, Chu CM, Cheng VC, Chan KS, Hung IF, Poon LL, Law KI, Tang BS, Hon TY, Chan CS, Chan KH. 2003. Clinical progression and viral load in a community outbreak of coronavirus-associated SARS pneumonia: a prospective study. The Lancet 361(9371):1767-72.
6. Zhao Z, Zhang F, Xu M, Huang K, Zhong W, Cai W, Yin Z, Huang S, Deng Z, Wei M, Xiong J. Description and clinical treatment of an early outbreak of severe acute respiratory syndrome (SARS) in Guangzhou, PR China. Journal of medical microbiology. 2003 Aug 1;52(8):715-20.
7. Wang JT, Sheng WH, Fang CT, Chen YC, Wang JL, Yu CJ, Chang SC, Yang PC. 2004. Clinical manifestations, laboratory findings, and treatment outcomes of SARS patients. Emerging Infect Dis 10(5):818.

S. Table 3. Case reports of patients with pulmonary virus infections, mainly MERS, and clinical interventions to prevent or cure secondary bacterial infections.

Total Patient number	Development of viral Pneumonia (n)	Development of secondary infection (n)	Prophylactic Antibiotics	Therapeutic Antibiotics	Fatality due to viral pneumonia (n)	Reference
N=12 (MERS-CoV)	12 (100%)	2 (16.6%) - MRSA and *S. pneumoniae*	Broad-spectrum antimicrobials	N/D	7 (58.3%)	(1)
N=5 (MERS-CoV)	5 (100%)	N/D	Levofloxacin (500 mg/2 days) and imipenem (250 mg twice daily) or levofloxacin (750 mg/2 days) and imipenem (500 mg/6 h) or levofloxacin (750 mg/2 days) and imipenem (250 mg/6 h) or imipenem (250 mg twice daily)	N/D	5 (100%)	(2)
N=70 (MERS-CoV)	63 (90%)	30 (42.9%) - carbapenem-resistant *A. baumannii*, VRE and MRSA	N/D	N/D	42 (60%)	(3)

Total Patient number	Development of viral Pneumonia (n)	Development of secondary infection (n)	Prophylactic Antibiotics	Therapeutic Antibiotics	Fatality due to viral pneumonia (n)	Reference
N=47 (MERS-CoV)	47 (100%)	N/D	Broad-spectrum antibiotics	N/D	28 (60%)	(4)
N=4 (MERS-CoV)	4 (100%)	N/D	Piperacillin-tazobactam, azithromycin, ceftriaxone and trimethoprim-sulfamethoxazole	N/D	2 (50%)	(5)

N/D - No data available; MRSA – Methicillin-resistant *Staphylococcus aureus*; VRE – Vancomycin-resistant Enterococci

References:

1. Arabi YM, Arifi AA, Balkhy HH, Najm H, Aldawood AS, Ghabashi A, Hawa H, Alothman A, Khaldi A, Al Raiy B. Clinical course and outcomes of critically ill patients with Middle East respiratory syndrome coronavirus infection. Annals of internal medicine. 2014 Mar 18;160(6):389-97.
2. Al-Tawfiq JA, Momattin H, Dib J, Memish ZA. Ribavirin and interferon therapy in patients infected with the Middle East respiratory syndrome coronavirus: an observational study. International Journal of Infectious Diseases. 2014 Mar 1;20:42-6.
3. Saad M, Omrani AS, Baig K, Bahloul A, Elzein F, Matin MA, Selim MA, Al Mutairi M, Al Nakhli D, Al Aidaroos AY, Al Sherbeeni N. Clinical aspects and outcomes of 70 patients with Middle East respiratory syndrome coronavirus infection: a single-center experience in Saudi Arabia. International Journal of Infectious Diseases. 2014 Dec 1;29:301-6.
4. Assiri A, Al-Tawfiq JA, Al-Rabeeah AA, Al-Rabiah FA, Al-Hajjar S, Al-Barrak A, Flemban H, Al-Nassir WN, Balkhy HH, Al-Hakeem RF, Makhdoom HQ. Epidemiological, demographic, and clinical characteristics of 47 cases of Middle East respiratory syndrome coronavirus disease from Saudi Arabia: a descriptive study. The Lancet infectious diseases. 2013 Sep 1;13(9):752-61.
5. Memish ZA, Zumla AI, Al-Hakeem RF, Al-Rabeeah AA, Stephens GM. Family cluster of Middle East respiratory syndrome coronavirus infections. New England Journal of Medicine. 2013 Jun 27;368(26):2487-94.

S. Table 4. Case reports of patients with pulmonary virus infections, mainly influenza virus, and clinical interventions to prevent or cure secondary bacterial infections.

Total Patient number	Development of viral Pneumonia (n)	Development of secondary infection (n)	Prophylactic Antibiotics	Therapeutic Antibiotics	Fatality due to viral pneumonia (n)	Reference
N=252 (H1N1)	136 (54%)	19 (7.5%)	Azithromycin (44%), ceftriaxone (37%), fluoroquinolone (36%), vancomycin (17%), cephalosporin (16%), 74% received two or more antibiotics	Azithromycin, ceftriaxone, fluoroquinolone, vancomycin, cephalosporin	11 (4%)	(1)
N=242 (H1N1)	165 (68%)	7 (2.8%) - *S. pneumoniae*, *P. aeruginosa*, *S. viridians* and *S. hominis*	217 (89%) Ceftriaxone, amoxiclavulanic, ampi sublactam, vancomycin, piperacillin-tazobactam and imipenem	N/D	81 (33%)	(2)

Total Patient number	Development of viral Pneumonia (n)	Development of secondary infection (n)	Prophylactic Antibiotics	Therapeutic Antibiotics	Fatality due to viral pneumonia (n)	Reference
N=174 (H1N1)	174 (100%)	79 (45.4%) - *S. aureus* (25, MRSA=19), *Streptococci* (10), *P. aeruginosa* (8), *Enterobacteriaceae* (5), and urinary tract infections: *Enterococci* (8), *Enterobacteriaceae* (8), *P. aeruginosa* (1)	Vancomycin (50.7%), cefepime (40.3%), azithromycin (40.3%), meropenem (23.9%), linezolid (20.9%), vancomycin plus cefepime (28.4%) and vancomycin plus meropenem (13.4%)	N/D	N/D	(3)
N=57 (38-H1N1; 9-H3N2; 8-Influenza B; 2 RSV)	57 (100%)	*S. pneumoniae* (n=46, 80.7%), *H. influenza* (n=4, 8.77%), *S. aureus* (n=5, 7.01%), *Chlamydophila pneumoniae* (n=1, 1.75%), *Moraxella catarrhalis* (n=1, 1.75%)	Beta-lactam monotherapy (14.03%), beta-lactam+fluoroquinolone (54.3%), fluoroquinolone (14.03%), beta-lactam+macrolide (1.75%) and broad-spectrum antibiotics (3.51%)	N/D	N/D	(4)
N=47 (H1N1)	N/D	13 (28%) - *S. pneumonia* (n=9, 19%), *S. pyogenes* (n=3, 6%) and one patient had both	N/D	N/D	N/D	(5)

Total Patient number	Development of viral Pneumonia (n)	Development of secondary infection (n)	Prophylactic Antibiotics	Therapeutic Antibiotics	Fatality due to viral pneumonia (n)	Reference
N=100 (H1N1)	N/D	26 (26%) - *S. pneumonia* (n=9), *S. pyogenes* (n=3), *S. pneumonia*/*S. pyogenes* (n=1), *S. pyogenes*/*S. mitis* (n=1), *S. mitis* (n=1), *S. agalactiae* (n=1), MRSA (n=4), MRSA/ *S. pyogenes* (n=1), MRSA/*H. influenza* (n=1) and MSSA (n=4)	N/D	N/D	N/D	(6)
N=20 (H1N1)	20 (100%)	4 (20%) - *S. pneumonia* (n=2) and *P. aeruginosa* (n=2)	N/D	N/D	20 (100%)	(7)
N=18 (H1N1)	18 (100%)	*A. baumannii*, *Achromobacter xylosoxidans*, MRSA or *E. coli*	Ceftriaxone (n=17) and clarithromycin (n=10), levofloxacin (n=3), vancomycin (n=7), cefepime (n=5), imipenem (n=5) and dicloxacillin (n=2)	N/D	7 (38.8%)	(8)

Total Patient number	Development of viral Pneumonia (n)	Development of secondary infection (n)	Prophylactic Antibiotics	Therapeutic Antibiotics	Fatality due to viral pneumonia (n)	Reference
N=29 (HcoVs- 12 OC43, 7 229E, 6 NL63, 4 HKU1)	13 (44%)	3 (10%) – 1 *P. aeruginosa*, 1 *E. coli* and 1 *S. aureus*	22/29 (95%)	N/D	3 (23%)	(9)
N=47* (Influenza virus)	10 (21%)	8 (17%) – 3 *Staphylococcus*, 3 group A streptococci, 1 *S. pneumoniae*, 1 *Bordetella pertussis*	N/D	N/D	47 (100%)	(10)
N=838 (pH1N1)	564 (67%)	274/838 (31.5%) – 71 *S. aureus* (34 MRSA), 30 *Pseudomonas*, 7 *S. pneumoniae*, 13 *H. influenza*, 7 *S. pyogenes*	Vancomycin	Vancomycin	75 (13%)	(11)

Total Patient number	Development of viral Pneumonia (n)	Development of secondary infection (n)	Prophylactic Antibiotics	Therapeutic Antibiotics	Fatality due to viral pneumonia (n)	Reference
N=153 (Influenza virus)	71 (46%)	24 (15.6%) – 11 *S. aureus* (6 MRSA), 1 *Staphylococcus*, 2 *S. pneumoniae*, 3 Group A streptococcus, 1 *B. pertussis*, 4 *H. influenzae*, 1 *P. aeruginosa*, 1 *E. faecalis*, 1 *N. meningitidis*, 1 *Mycoplasma pneumoniae*	N/D	N/D	71* (100%)	(12)
N=2992 (Influenza virus)	1072 (35.8%)	21 (1%) *S. aureus*	N/D	N/D	10 (1%)	(13)
N=272 (H1N1)	100 (40%)	3 (1%) – 1 *E. coli*, 1 *S. pneumoniae*, 1 *S. aureus*	206 (75.7%%) – ceftriaxone (94 patients), azithromycin (84 patients),vancomycin (56 patients), levofloxacin (47 patients)	N/D	19 (19%)	(14)

N/D - No data available; MRSA – Methicillin-resistant *Staphylococcus aureus*; *Postmortem studies of the influenza infected children (<18 years)

References:

1. Chitnis AS, Truelove SA, Druckenmiller JK, Heffernan RT, Davis JP. Epidemiologic and clinical features among patients hospitalized in Wisconsin with 2009 H1N1 influenza A virus infections, April to August 2009. Wisconsin Medical Journal (WMJ). 2010 Aug;109(4):201.
2. Kusznierz G, Uboldi A, Sosa G, Torales S, Colombo J, Moyano C, Escobar H, Lejona S, Anchart E, Gómez A, Imaz S. Clinical features of the hospitalized patients with 2009 pandemic influenza A (H1N1) in Santa Fe, Argentina. Influenza and other respiratory viruses. 2013 May;7(3):410-7.
3. Crotty MP, Meyers S, Hampton N, Bledsoe S, Ritchie DJ, Buller RS, Storch GA, Kollef MH, Micek ST. Impact of antibacterials on subsequent resistance and clinical outcomes in adult patients with viral pneumonia: an opportunity for stewardship. Critical Care. 2015 Dec;19(1):404.
4. Abelenda-Alonso G, Rombauts A, Gudiol C, Meije Y, Ortega L, Clemente M, Ardanuy C, Niubó J, Carratalà J. Influenza and bacterial coinfection in adults with community-acquired pneumonia admitted to conventional wards: Risk factors, clinical features, and outcomes. InOpen Forum Infectious Diseases 2020 Mar (Vol. 7, No. 3, p. ofaa066). US: Oxford University Press.
5. Hughes JM, Wilson ME, Hughes JM, Wilson ME, Lee EH, Wu C, Lee EU, Stoute A, Hanson H, Cook HA, Nivin B. Fatalities associated with the 2009 H1N1 influenza A virus in New York city. Clinical Infectious Diseases. 2010 Jun 1;50(11):1498-504.
6. Shieh WJ, Blau DM, Denison AM, DeLeon-Carnes M, Adem P, Bhatnagar J, Sumner J, Liu L, Patel M, Batten B, Greer P. 2009 pandemic influenza A (H1N1): pathology and pathogenesis of 100 fatal cases in the United States. The American journal of pathology. 2010 Jul 1;177(1):166-75.
7. Nakajima N, Sato Y, Katano H, Hasegawa H, Kumasaka T, Hata S, Tanaka S, Amano T, Kasai T, Chong JM, Iiduka T. Histopathological and immunohistochemical findings of 20 autopsy cases with 2009 H1N1 virus infection. Modern Pathology. 2012 Jan;25(1):1-3.
8. Perez-Padilla R, De La Rosa-Zamboni D, Ponce de Leon S, Hernandez M, Quiñones-Falconi F, Bautista E, Ramirez-Venegas A, Rojas-Serrano J, Ormsby CE, Corrales A, Higuera A. Pneumonia and respiratory failure from swine-origin influenza A (H1N1) in Mexico. New England journal of medicine. 2009 Aug 13;361(7):680-9.
9. Garbino J, Crespo S, Aubert JD, Rochat T, Ninet B, Deffernez C, Wunderli W, Pache JC, Soccal PM, Kaiser L. 2006. A prospective hospital-based study of the clinical impact of non–severe acute respiratory syndrome (non-SARS)–related human coronavirus infection. Clin Infect Dis 43(8):1009-15
10. Guarner J, Paddock CD, Shieh WJ, Packard MM, Patel M, Montague JL, Uyeki TM, Bhat N, Balish A, Lindstrom S, Klimov A. 2006. Histopathologic and immunohistochemical features of fatal influenza virus infection in children during the 2003–2004 season. Clin Infect Dis 43(2):132-40
11. Randolph AG, Vaughn F, Sullivan R, Rubinson L, Thompson BT, Yoon G, Smoot E, Rice TW, Loftis LL, Helfaer M, Doctor A. 2011. Critically ill children during the 2009–2010 influenza pandemic in the United States. Pediatrics 128(6):e1450-8.
12. Bhat N, Wright JG, Broder KR, Murray EL, Greenberg ME, Glover MJ, Likos AM, Posey DL, Klimov A, Lindstrom SE, Balish A. 2005. Influenza-associated deaths among children in the United States, 2003–2004. N Eng J Med 353(24): 2559-67.
13. Dawood FS, Fiore A, Kamimoto L, Nowell M, Reingold A, Gershman K, Meek J, Hadler J, Arnold KE, Ryan P, Lynfield R. 2010. Influenza-associated pneumonia in children hospitalized with laboratory-confirmed influenza, 2003–2008. The Pediatric Infect Dis J 29(7):585.

14. Jain S, Kamimoto L, Bramley AM, Schmitz AM, Benoit SR, Louie J, Sugerman DE, Druckenmiller JK, Ritger KA, Chugh R, Jasuja S. 2009. Hospitalized patients with 2009 H1N1 influenza in the United States, April–June 2009. N Eng J Med 361(20):1935-44.
