# Supplementary material for: Secondary Bacterial Infections in Patients With Viral Pneumonia
Source: Front Med (Lausanne). 2020 Aug 5;7:420. doi: 10.3389/fmed.2020.00420 (PMC7419580; doi:10.3389/fmed.2020.00420)
Supplement: Supplemental Data Sheet 1 — Case reports of patients with secondary bacterial infections. [file Data_Sheet_1.pdf]

A. Case reports of antibacterial therapy in COVID-19 patients

1. Wang D *et al*., 2020, report a study conducted in Zhongnan Hospital (Wuhan, China) of 138 hospitalized patients with Novel Coronavirus-infected Pneumonia (NCIP) (28). As a prophylactic measure, all patients (n=138) received antibiotics: moxifloxacin (n=89, 64.4%), ceftriaxone (n=34, 24.6%), and azithromycin (n=25, 18.1%). The severity of bacterial infections post-treatment was not reported.

2. Holshue ML *et al*., 2020, describe the first case of COVID-19 in the United States (Washington State), a 35-year-old male patient admitted with the symptoms of cough and fever (29). Considering the clinical condition and to avoid hospital-acquired pneumonia, vancomycin (1g/ 8 hours) and cefepime (1g/ 8 hours) were administered intravenously, as a prophylactic measure. The patient recovered and antibiotics were discontinued.

3. Guan WJ *et al*., 2020, provide clinical data of 1099 patients with COVID-19 from 30 provinces in China (30). 58% of the patients (n=637) received intravenous antibiotics to prevent bacterial infections. No details on the antibiotic compounds or their dosage were provided in this report. Of 173 disease severity patients, 139 (80.3%) received intravenous antibiotics. No data was provided with regards to secondary bacterial infections.

4. Yang X *et al*., 2020, published a single-centered, retrospective, observational study that included 52 critically ill adult patients with COVID-19 at Jin Yin-tan hospital (Wuhan, China) (26). Patients were admitted to the ICU with pneumonia-like symptoms. Antibacterial therapy was given to 49/52 (94%) patients as a precaution, and hospital-acquired infections developed in 7 (13.5%) patients. No details on the antibiotic compounds or their dosage were provided in this report. One patient was infected with each of carbapenem-resistant *K. pneumoniae* (pulmonary and blood stream infection), Extended-Spectrum Beta-Lactamase (ESBL)-positive *K. pneumoniae*, ESBL-positive *P. aeruginosa*, ESBL-negative *Serratia marcescens*, *Aspergillus flavus, A. fumigatus,* and *Candida albicans* in the urine sample of one

patient. Two of 6 patients with hospital-acquired pneumonia died, as well as two patients with bacteraemia and UTI, respectively.

5. Zhou F *et al*., 2020, report a retrospective, multi-centered cohort study of 191 patients from two hospitals (Jin Yin-tan Hospital and Wuhan Pulmonary Hospital, China) (27). 181 patients (95%) received prophylactic antibiotic therapy. No details on the antibiotic compounds or their dosage were provided in this report. Nearly 60% of the patients (mostly adults) developed sepsis. Secondary infections were diagnosed with the signs of pneumonia or bacteraemia, mainly ventilator-associated and hospital-acquired infections. Half of the fatalities (27/54) experienced secondary infections, and ventilator-associated pneumonia occurred in 10 patients. In total, 137 were discharged and 54 patients died in hospital.

6. Ruan Q *et al*., 2020, report data of a retrospective study of 150 patients in two hospitals (JinYin-tan Hospital and Tongji Hospital, China) with 68 recorded deaths and 82 cases that recovered and were discharged (31). Sixteen percent (11/68) of fatalities experienced bacterial secondary infections while one patient was discharged after having had secondary infections. No detail was provided on any antibacterial treatment in this report. The authors concluded that one reason for fatal outcomes in COVID-19 cases was due to the presence of secondary bacterial infections.

7. Wu C *et al*., 2020, published a retrospective cohort study of 201 patients with confirmed COVID-19 pneumonia at Jin Yin-tan Hospital, China (32). Among 201 patients, 196 (97.5%) received prophylactic antibiotic treatment. No details on the antibiotic compounds or their dosage were provided in this report. Possible bacterial/ fungal infections (sputum samples) were detected in 148 (73.6%) patients, showing that most patients acquired secondary infections, needing further targeted treatment. In total, 43/84 patients who received antibiotic therapy and developed Acute Respiratory Distress Syndrome (ARDS) died.

8. Chen N *et al*., 2020, describe a retrospective, single-center study in Wuhan Jinyintan Hospital that included 99 patients with pneumonia, of which 49 were linked to the Huanan seafood market (25). Antibiotic therapy was given to 70/99 (71%) patients, of

which 25 (25%) patients were treated with a single antibiotic and 45 (45%) with combination therapy, detailed below. No detailed information is provided regarding the outcome of the prophylactic antibiotic therapy, but 17% of severely ill patients acquired bacterial co-infections, likely resistant to the administered drugs. One (1%) patient acquired ventilator-associated pneumonia with multiple bacterial strains including MRSA, *A. baumannii* and *K. pneumoniae*. Antibiotics of the classes of cephalosporins, quinolones, carbapenems, and tigecycline were used for the therapy against MRSA. A strain of *A. baumannii* isolated from the same patient was found to be highly resistant to all used antibiotics.

9. Huang C *et al*., 2020, published a single-center study of 41 confirmed COVID-19 cases admitted to a hospital in Wuhan, China (33). All patients had pneumonia and underwent prophylactic antibiotic therapy. No details on the antibiotic compounds or their dosage were provided in this report. Thirteen of 41 (32%) patients were admitted to the ICU and four (31%) developed secondary bacterial infections, nosocomial pneumonia or bacteremia. No data with regards to the severity of bacterial infections were provided in this study.

10. Wu J *et al*., 2020, report a retrospective study including 80 patients from the First People's Hospital of Yancheng City, the second People's Hospital of Yancheng City, and the Fifth People's Hospital of Wuxi in China (34). All patients were confirmed to have COVID-19, and 73 (91%) patients were treated with a single antibiotic, moxifloxacin, as a prophylactic measure. Twenty-one patients were discharged and none developed secondary infections. No information regarding bacterial co- or secondary infections was provided for the remaining 59 patients.

11. Gautret P *et al*., 2020, describe an open-label non-randomized clinical trial for the treatment of twenty COVID-19 patients with hydroxychloroquine targeting the virus, in combination with the antibiotic compound azithromycin (35). Six patients with pneumonia received azithromycin (500 mg on day 1 followed by 250 mg / day for the next four days) to prevent bacterial infections, under electrocardiogram control as the antibiotic can potentially cause fatal irregular heart rhythm. All patients treated with the combination fully recovered and were negative for SARS-CoV-2 within five days

of the treatment. The authors suggest a synergistic effect of the combination of hydroxychloroquine together with azithromycin. Azithromycin has been shown to be active against Zika and Ebola viruses *in vitro*, in addition to the compound's activity on bacteria (35,36).

12. Easom et al., 2020, reported a series of 68 patients assessed at a Regional Infection Unit in the UK (36). Of the 68 confirmed COVID-19 patients, 8 (11.7%) patients were treated with prophylactic antibiotics doxycycline (n=5, 62.5%) and moxifloxacin (n=3, 37.5%). Further laboratory investigation showed that one patient was infected with *S. aureus* (MSSA), one with *E. coli* and *H. influenza* and two with *H. influenza*. No reports about the antibiotic treatment. It was recorded that the number of patients (14/68) requiring antibiotic therapy had complications during admission.

13. Chen et al., 2020, conducted a retrospective case study that included 799 patients at the Tongji Hospital, China (37). The study included 113 (14.1%) deceased patients, 161 (20.1%) discharged patients and 525 (65.7%) patients were still in medical observation. In total, 105 (95%) deceased patients and 144 (89%) recovered patients received prophylactic antibiotic therapy using moxifloxacin, cefoperazone and azithromycin. No laboratory investigated bacterial identification was reported. The study concluded that one third of the deceased patients might have developed secondary bacterial infection which is undetected and for the patients without bacterial infections, prophylactic antibiotic therapy is ineffective.

14. Wang et al., 2020, analysed 69 SARS-CoV-2 infected patients who were hospitalized in Union Hospital, Wuhan, China (38). 66/69 (95.6%) patients received prophylactic antibiotic therapy, of which 38 patients received moxifloxacin. During the course of the treatment, *E. cloacae* (n=2) and *A. baumannii* (n=3) infections were observed. No reports about the antibiotic treatment and the severity of bacterial infections.

15. Wang et al., 2020, reported 125 patients, including 25 critical patients in Fuyang, Anhui province, China (39). All the 125 patients (100%) received prophylactic antibiotic therapy. Of which 71 (56.8%) patients developed secondary bacterial infections, therefore treated with antibiotics. The most common complication was due

to secondary infections and 6/71 (8.4%) patients developed ARDS. No data was available about the type of antibiotics used and the bacterial infection.

References:

28. Wang D, Hu B, Hu C, Zhu F, Liu X, Zhang J, Wang B, Xiang H, Cheng Z, Xiong Y, Zhao Y. 2020. Clinical characteristics of 138 hospitalized patients with 2019 novel coronavirus–infected pneumonia in Wuhan, China. Jama 323(11):1061-1069.
29. Holshue ML, DeBolt C, Lindquist S, Lofy KH, Wiesman J, Bruce H, Spitters C, Ericson K, Wilkerson S, Tural A, Diaz G. 2020. First case of 2019 novel coronavirus in the United States. N Engl J Med 382:929-936.
30. Guan WJ, Ni ZY, Hu Y, Liang WH, Ou CQ, He JX, Liu L, Shan H, Lei CL, Hui DS, Du B. 2020. Clinical characteristics of coronavirus disease 2019 in China. N Engl J Med.
26. Yang X, Yu Y, Xu J, Shu H, Liu H, Wu Y, Zhang L, Yu Z, Fang M, Yu T, Wang Y. 2020. Clinical course and outcomes of critically ill patients with SARS-CoV-2 pneumonia in Wuhan, China: a single-centered, retrospective, observational study. The Lancet Respir Med. In-press
27. Zhou F, Yu T, Du R, Fan G, Liu Y, Liu Z, Xiang J, Wang Y, Song B, Gu X, Guan L. 2020. Clinical course and risk factors for mortality of adult inpatients with COVID-19 in Wuhan, China: a retrospective cohort study. The Lancet 395(10229): 1054-1062.
31. Ruan Q, Yang K, Wang W, Jiang L, Song J. 2020. Clinical predictors of mortality due to COVID-19 based on an analysis of data of 150 patients from Wuhan, China. Intensive Care Med 3:1-3.
32. Wu C, Chen X, Cai Y, Zhou X, Xu S, Huang H, Zhang L, Zhou X, Du C, Zhang Y, Song J. 2020. Risk factors associated with acute respiratory distress syndrome and death in patients with coronavirus disease 2019 pneumonia in Wuhan, China. JAMA Intern Med.
25. Chen N, Zhou M, Dong X, Qu J, Gong F, Han Y, Qiu Y, Wang J, Liu Y, Wei Y, Yu T. 2020. Epidemiological and clinical characteristics of 99 cases of 2019 novel coronavirus pneumonia in Wuhan, China: a descriptive study. The Lancet 395(10223):507-13.
33. Huang C, Wang Y, Li X, Ren L, Zhao J, Hu Y, Zhang L, Fan G, Xu J, Gu X, Cheng Z. 2020. Clinical features of patients infected with 2019 novel coronavirus in Wuhan, China. The Lancet 395(10223):497-506.
34. Wu J, Liu J, Zhao X, Liu C, Wang W, Wang D, Xu W, Zhang C, Yu J, Jiang B, Cao H. 2020. Clinical Characteristics of Imported Cases of COVID-19 in Jiangsu Province: A Multicenter Descriptive Study. Clin Infect Dis.
35. Gautret P, Lagier JC, Parola P, Meddeb L, Mailhe M, Doudier B, Courjon J, Giordanengo V, Vieira VE, Dupont HT, Honoré S. 2020. Hydroxychloroquine and azithromycin as a treatment of COVID-19: results of an open-label non-randomized clinical trial. Int J Antimicrob Agents: 105949.
36. Easom N, Moss P, Barlow G, Samson A, Taynton T, Adams K, Ivan M, Burns P, Gajee K, Eastick K, Lillie PJ. 68 Consecutive patients assessed for COVID−19 infection; experience from a UK regional infectious disease unit. Influenza and Other Respiratory Viruses. 2020 Jan 1.
37. Chen T, Wu D, Chen H, Yan W, Yang D, Chen G, Ma K, Xu D, Yu H, Wang H, Wang T. Clinical characteristics of 113 deceased patients with coronavirus disease 2019: retrospective study. Bmj. 2020 Mar 26;368.
38. Wang Z, Yang B, Li Q, Wen L, Zhang R. Clinical features of 69 cases with coronavirus disease 2019 in Wuhan, China. Clinical Infectious Diseases. 2020 Mar 16.
39. Wang R, Pan M, Zhang X, Fan X, Han M, Zhao F, Miao M, Xu J, Guan M, Deng X, Chen X. Epidemiological and clinical features of 125 Hospitalized Patients with COVID-19 in Fuyang, Anhui, China. International Journal of Infectious Diseases. 2020 Apr 11.

B. Case reports of patients with SARS-related Secondary Bacterial Infections and Treatment

1. Chong *et al*., 2004, analysed the deaths caused by the SARS epidemic in Singapore (45). The tested tissues samples taken during autopsy, and could detect bacterial infections in 50% of the cases; out of the 14 patients, 7 developed secondary bacterial infections, in four no infection was observed, while data for another three was not reported. The infections were caused by *P. aeruginosa*, MRSA, *Streptococcus* spp., *Klebsiella* spp., *Enterococcus* spp., *Acinetobacter baumannii* and *Enterobacter* spp. The clinical data during the patients' treatment, such as records of any antibiotics administered are not provided in this study.

2. So *et al*., 2003, studied a group of 31 SARS patients for a standard treatment protocol at Pamela Youde Nethersole Eastern Hospital in Hong Kong (46). All the patients were treated prophylactically with levofloxacin (500 mg/day) or clarithromycin (500 mg twice daily), and amoxicillin and clavulanic acid (375 mg thrice daily). Piperacillin and tazobactum was given to seven patients who had developed fever and elevated white blood cell counts. None of the patients had positive bacterial cultures after the antibiotic treatment and no fatalities were reported.

3. Lee *et al*., 2003, performed a cohort study that included 138 patients at Prince of Wales hospital in Hong Kong (47). Five patients (3.6%) were found to have secondary bacterial infections caused by *H. influenza* (n=3), *S. pneumoniae* (n=1) and *K. pneumoniae* (n=1). One of the five seriously ill ICU patients was treated with amoxicillin-clavulanate and clarithromycin. No record for the other patients or details about the prophylactic antibiotic therapy was provided.

4. Tsang *et al*., 2003, studied the clinical treatments and disease outcomes of 10 patients in Hong Kong hospitals (48). All the patients received beta-lactam antibiotics (augmentin, rocephin or maxipime) and a macrolide (clarithromycin or azithromycin) twice daily for four days. None of the patients developed complications due to secondary bacterial infections during the period of this study.

5. Peiris *et al.*, 2003, performed a follow-up study of 75 patients admitted to the United Christian Hospital, Hong Kong (49). All the patients were treated with amoxicillin-clavulanate (1.2 g/8 h) and azithromycin (500 mg/day) or levofloxacin (500 mg/24 h). 10 patients (13.3%) developed sepsis, with three suffering from bacteraemia caused by *Stenotrophomonas maltophilia*. Four had catheter-related sepsis, and three had nosocomial pneumonia due to *K. pneumoniae* (n=2) and *E. coli* (n=1).

6. Zhao *et al.*, 2003, studied the clinical symptoms of 190 SARS patients in Guangdong, China (50). Forty cases were treated with cefoperazone/sulbactam (2 g twice daily), thirty with fluoroquinolone plus azithromycin (0.4 g/day), sixty cases with quinolone plus azithromycin (0.4 g/day) and another sixty cases with levofloxacin (0.2 g twice daily) plus azithromycin (0.6 g/day). None of the patients developed bacterial infections during the prophylactic antibiotic treatment.

7. Wang *et al.*, 2004, conducted a prospective study on 76 patients at National Taiwan University Hospital, in Taipei City (51). All the patients were treated prophylactically with moxifloxacin alone or ceftriaxone plus azithromycin. Nosocomial infections were detected in 18 patients, of which 6 were bloodstream infections caused by enterococci (n=3), MRSE (n=2) and MRSA (n=1). Other infections include *A. baumannii* (n=3), MRSA (n=4), MRSE (n=2) and *Enterococci* (n=3). The overall death rate was 19.7% (15/76) and 11 patients with underlying bacterial infections were classified as rapidly fatal.

References:

45. Chong PY, Chui P, Ling AE, Franks TJ, Tai DY, Leo YS, Kaw GJ, Wansaicheong G, Chan KP, Ean Oon LL, Teo ES. Analysis of deaths during the severe acute respiratory syndrome (SARS) epidemic in Singapore: challenges in determining a SARS diagnosis. Archives of pathology & laboratory medicine. 2004 Feb;128(2):195-204.
46. So LK, Lau AC, Yam LY, Cheung TM, Poon E, Yung RW, Yuen KY. Development of a standard treatment protocol for severe acute respiratory syndrome. The Lancet. 2003 May 10;361(9369): 1615-7.
47. Lee N, Hui D, Wu A, Chan P, Cameron P, Joynt GM, Ahuja A, Yung MY, Leung CB, To KF, Lui SF. A major outbreak of severe acute respiratory syndrome in Hong Kong. New England Journal of Medicine. 2003 May 15;348(20):1986-94.
48. Tsang KW, Ho PL, Ooi GC, Yee WK, Wang T, Chan-Yeung M, Lam WK, Seto WH, Yam LY, Cheung TM, Wong PC. A cluster of cases of severe acute respiratory syndrome in Hong Kong. New England Journal of Medicine. 2003 May 15;348(20):1977-85.

49. Peiris JS, Chu CM, Cheng VC, Chan KS, Hung IF, Poon LL, Law KI, Tang BS, Hon TY, Chan CS, Chan KH. 2003. Clinical progression and viral load in a community outbreak of coronavirus-associated SARS pneumonia: a prospective study. The Lancet 361(9371):1767-72.
50. Zhao Z, Zhang F, Xu M, Huang K, Zhong W, Cai W, Yin Z, Huang S, Deng Z, Wei M, Xiong J. Description and clinical treatment of an early outbreak of severe acute respiratory syndrome (SARS) in Guangzhou, PR China. Journal of medical microbiology. 2003 Aug 1;52(8):715-20.
51. Wang JT, Sheng WH, Fang CT, Chen YC, Wang JL, Yu CJ, Chang SC, Yang PC. 2004. Clinical manifestations, laboratory findings, and treatment outcomes of SARS patients. Emerging Infect Dis 10(5):818.

C. Case reports of patients with MERS-related Secondary Bacterial Infections and Treatment

1. Arabi *et al*., 2012, studied the clinical outcomes of 12 patients with confirmed MERS-CoV infection in two tertiary care hospitals in Saudi Arabia (55). All the patients received prophylactic antibiotic therapy using broad-spectrum antimicrobials. No data was reported about the type of antibiotics used. Two patients were co-infected with MRSA and *S. pneumoniae* during their treatment in the ICU. The clinical outcomes of the bacterial infections were not recorded.

2. Al-Tawfiq *et al*., 2014, performed a retrospective observational study on five MRES patients in Saudi Arabia (56). Case 1: A 62-year-old woman was treated with levofloxacin (500 mg/2 days) for 15 days and imipenem (250 mg twice daily) for 3 days. Case 2: A 58-year-old man was treated with levofloxacin (500 mg/2 days) for 6 days and imipenem (250 mg twice daily) for 7 days. Case 3: A 63-year-old woman was treated with levofloxacin (750 mg/2 days) for 7 days and imipenem (500 mg/6 h) for 8 days. Case 4: A 81-year-old man was treated with levofloxacin (750 mg/2 days) for 5 days and imipenem (250 mg/6 h) for 6 days. Case 5: A 24-year-old man was treated with imipenem (250 mg twice daily) for 11 days. All the 5 cases died of multi-organ failure but no data was collected about the bacterial infections during autopsy.

3. Saad *et al*., 2014, performed a retrospective study on 70 patients in Saudi Arabia (57). No data was provided about the antibiotic therapy. Concomitant infections were reported in 30 patients (42.9%) that included bacteremia, bacterial pneumonia, UTI, skin and soft tissue infection and candidemia. Multi-drug resistant bacteria were isolated from 22 patients (31.4%), including carbapenem-resistant *A. baumannii*, VRE and MRSA. The

majority (15/22) of the bacterial infections happened in ICU care and no reports on the antibiotic treatment and patient outcomes.

4. Assiri *et al.*, 2013, did a descriptive study that includes 47 cases in Saudi Arabia (58). Of the 47 patients, 42 were critically ill, requiring ICU. All the patients were treated with broad-spectrum antibiotics but no data was provided about the antibiotic therapy, regarding type and dosage. None of the patients were tested positive for bacterial infections with 3 days of admission but no microbiological examination was performed for community-acquired pneumonia.

5. Memish *et al.*, 2013, studied the family cluster of 4 patients in Riyadh (59). Patient 1 was treated with the broad-spectrum antibiotics piperacillin-tazobactam, azithromycin, and trimethoprim-sulfamethoxazole but died of septic shock. Patient 2 was treated with azithromycin and ceftriaxone but died of treatment failure as bacteria were resistant to the used drugs. Patient 3 and 4 were treated with azithromycin and ceftriaxone and recovered from MERS; the antibiotic therapy was continued for the next 5 days. The patients had no bacterial infections during the course of therapy.

References:

55. Arabi YM, Arifi AA, Balkhy HH, Najm H, Aldawood AS, Ghabashi A, Hawa H, Alothman A, Khaldi A, Al Raiy B. Clinical course and outcomes of critically ill patients with Middle East respiratory syndrome coronavirus infection. Annals of internal medicine. 2014 Mar 18;160(6):389-97.
56. Al-Tawfiq JA, Momattin H, Dib J, Memish ZA. Ribavirin and interferon therapy in patients infected with the Middle East respiratory syndrome coronavirus: an observational study. International Journal of Infectious Diseases. 2014 Mar 1;20:42-6.
57. Saad M, Omrani AS, Baig K, Bahloul A, Elzein F, Matin MA, Selim MA, Al Mutairi M, Al Nakhli D, Al Aidaroos AY, Al Sherbeeni N. Clinical aspects and outcomes of 70 patients with Middle East respiratory syndrome coronavirus infection: a single-center experience in Saudi Arabia. International Journal of Infectious Diseases. 2014 Dec 1;29:301-6.
58. Assiri A, Al-Tawfiq JA, Al-Rabeeah AA, Al-Rabiah FA, Al-Hajjar S, Al-Barrak A, Flemban H, Al-Nassir WN, Balkhy HH, Al-Hakeem RF, Makhdoom HQ. Epidemiological, demographic, and clinical characteristics of 47 cases of Middle East respiratory syndrome coronavirus disease from Saudi Arabia: a descriptive study. The Lancet infectious diseases. 2013 Sep 1;13(9):752-61.
59. Memish ZA, Zumla AI, Al-Hakeem RF, Al-Rabeeah AA, Stephens GM. Family cluster of Middle East respiratory syndrome coronavirus infections. New England Journal of Medicine. 2013 Jun 27;368(26):2487-94.

D. Case reports of patients with Influenza Viruses and Secondary Bacterial Infections

1. Chitnis et al., 2010, performed a prospective surveillance of H1N1 patients between April-August 2009 (65). Out of 252 patients, 204 (81%) received antibiotic therapy which includes azithromycin (44%), ceftriaxone (37%), fluoroquinolone (36%), vancomycin (17%), cephalosporin (16%), 74% received two or more antibiotics. During treatment positive bacterial cultures were obtained in 19 (7.5%) patients. Same antibiotics were used to treat bacterial infections and 11 (4%) died due to treatment failure or viral pneumonia.

2. Kusznierz et al., 2013, conducted a retrospective study among 242 H1N1 infected patients between May-July 2009 (66). The antibiotics used for prophylactic therapy and for treatment includes ceftriaxone, amoxiclavulanic, ampi sulbactam, vancomycin, piperacillin-tazobactam and imipenem. 89% (n=217) of patients received antibiotic therapy, 32.6% (n=79) received antibiotics before admission and 40% (n=97) received more than one antibiotic. Secondary bacterial infections were detected in seven (2.9%) patients and the pathogens identified were *S. pneumoniae*, *P. aeruginosa*, *S. viridians* and *S. hominis*. In total, 81 (33.4%) were fatal cases and 161 (66.5%) survived.

3. Crotty et al., 2015, performed a single-center retrospective cohort study between March 2013 and November 2014 (67). The study included 174 patients with viral pneumonia of which 79 (45.4%) had mixed viral-bacterial infection. The identified bacterial infections included co-respiratory infections: *S. aureus* (n=25, MRSA=19), *Streptococci* (n=10), *P. aeruginosa* (n=8), *Enterobacteriaceae* (n=5), and urinary tract infections: *Enterococci* (n=8), *Enterobacteriaceae* (n=8), *P. aeruginosa* (n=1), and skin infection (n=8), and Intra-abdominal infection (n=2). The most frequently used prophylactic antibiotics in patients without bacterial co-infection were vancomycin (50.7%), cefepime (40.3%), azithromycin (40.3%), meropenem (23.9%), linezolid (20.9%), vancomycin plus cefepime (28.4%) and vancomycin plus meropenem (13.4%). A total of 44 (65.7%) patients received MRSA coverage treatment with vancomycin or linezolid. The most common multi-drug resistant isolates identified were VRE (35%), coagulase negative *Staphylococcus* sp. (15%), *Escherichia coli* (10%), *Enterobacter cloacae* (10%), and *Stenotrophomonas maltophilia* (10%) and others included *K. pneumonia*e, *S. marcescens*, *S. pneumoniae*, and *Achromobacter* sp. This study concluded that the patients with viral pneumonia

might develop multi-drug resistant bacterial infections due to the long-course antibiotic therapy.

4. Abelenda-Alonso et al., 2020, documented a cohort study of adults admitted to conventional wards (two tertiary care hospitals in Barcelona, Spain) with community-acquired pneumonia (68). The study recorded 1123 episodes: 57 (5.1%) viral-bacterial co-infections, 98 (8.7%) viral infections and 968 (86.1%) bacterial infections were analysed in this study. 36.77% of the patients suffered from co-infections with bacterial and viral involvement. For 57 patients with viral-bacterial co-infections, the prophylactic treatments antibiotics (n=57) that were used were beta-lactam monotherapy (n=8, 14.03%), beta-lactam together with fluoroquinolone (n=31, 54.3%), fluoroquinolone alone (n=8, 14.03%), beta-lactam together with macrolide (n=1, 1.75%) and broad-spectrum antibiotics (n=2, 3.51%). Among the 57 patients with viral-bacterial co-infections, most of the viral infections were caused by Influenza A H1N1 (n=38, 66.6%). Less common were the Influenza A strain H3N2 (n=9, 15.7%) and Influenza B (n=8, 14.03%). In two cases an infection with the Respiratory syncytial virus was observed (n=2, 3.5%). The most common bacterial species found in the patients was *S. pneumoniae* (n=46, 80.7%), while the less commonly found bacteria belonged to strains of *H. influenza* (n=5, 8.77%), *S. aureus* (n=5, 7.01%), *Chlamydophila pneumoniae* (n=1, 1.75%), and *Moraxella catarrhalis* (n=1, 1.75%). In the cases of bacterial community-acquired pneumonia, along with the above mentioned bacteria some infections were caused by multiple pathogens and the presence of *Legionella* spp., *Coxiella burnetti*, *P. aeruginosa* was detected. This study concluded that the viral-bacterial co-infections are common among the younger patients (<12 years old) with morbidities and is one of the important factors that contribute to disease progression and therefore should be closely monitored during the treatment.

5. Hughes et al., 2010, reported clinical complications of 47 fatal cases due to 2009 H1N1 influenza between April and July 2009 in New York City, USA (69). It was found that 13 (28%) of the fatalities were due to bacterial co-infections which included *S. pneumonia* (n=9, 19%), *S. pyogenes* (n=3, 6%) and with one patient being infected by both pathogens. The study does not provide any information if any antibiotic treatment was

done. This study concludes that prophylactic antibiotic therapy could prove advantageous for patients with severe influenza infections.

6. Shieh et al., 2010, detailed clinical observations of one hundred patients with fatal 2009 H1N1 influenza infection during May to October 2009 in the USA (70). No information is provides in this report if prophylactic antibiotics were administered. In total, 26 (26%) were confirmed to have bacterial co-infections. The most frequently identified bacteria belonged to *S. pneumonia* (n=9), *S. pyogenes* (n=3), *S. pneumonia*/*S. pyogenes* (n=1), *S. pyogenes*/*S. mitis* (n=1), *S. mitis* (n=1), *S. agalactiae* (n=1), MRSA (n=4), MRSA/*S. pyogenes* (n=1), MRSA/*H. influenza* (n=1) and MSSA (n=4).

7. Nakajima et al., 2011, examined the 20 fatal cases with 2009 H1N1 influenza virus between August 2009 and February 2010 in Japan (71). No information is provided whether antibiotics were administered during the infection. Of the 20 cases, microbiological sampling was done in 11 cases. The bacteria identified were *S. pneumonia* (n=2) and *P. aeruginosa* (n=2) while no other bacterial pathogens were found.

8. Perez-Padilla et al., 2009, reported clinical outcomes of 18 patients that were infected with the 2009 H1N1 influenza virus during March to April 2009 in Mexico (72). All the admitted patients were treated with the antibiotics ceftriaxone (n=17) and clarithromycin (n=10). Some patients were given additional antibiotics: Levofloxacin (n=3), vancomycin (n=7), cefepime (n=5), imipenem (n=5) and dicloxacillin (n=2). Four patients were diagnosed with ventilator-associated pneumonia caused by *A. baumannii*, *Achromobacter xylosoxidans*, MRSA or *E. coli*. Seven of 18 patients died due to viral pneumonia. The study recorded the development of resistant bacterial infections in four patients despite the prophylactic antibiotic therapy.

References:

65. Chitnis AS, Truelove SA, Druckenmiller JK, Heffernan RT, Davis JP. Epidemiologic and clinical features among patients hospitalized in Wisconsin with 2009 H1N1 influenza A virus infections, April to August 2009. Wisconsin Medical Journal (WMJ). 2010 Aug;109(4):201.
66. Kusznierz G, Uboldi A, Sosa G, Torales S, Colombo J, Moyano C, Escobar H, Lejona S, Anchart E, Gómez A, Imaz S. Clinical features of the hospitalized patients with 2009 pandemic

influenza A (H1N1) in Santa Fe, Argentina. Influenza and other respiratory viruses. 2013 May; 7(3):410-7.

67. Crotty MP, Meyers S, Hampton N, Bledsoe S, Ritchie DJ, Buller RS, Storch GA, Kollef MH, Micek ST. Impact of antibacterials on subsequent resistance and clinical outcomes in adult patients with viral pneumonia: an opportunity for stewardship. Critical Care. 2015 Dec;19(1): 404.
68. Abelenda-Alonso G, Rombauts A, Gudiol C, Meije Y, Ortega L, Clemente M, Ardanuy C, Niubó J, Carratalà J. Influenza and bacterial coinfection in adults with community-acquired pneumonia admitted to conventional wards: Risk factors, clinical features, and outcomes. InOpen Forum Infectious Diseases 2020 Mar (Vol. 7, No. 3, p. ofaa066). US: Oxford University Press.
69. Hughes JM, Wilson ME, Hughes JM, Wilson ME, Lee EH, Wu C, Lee EU, Stoute A, Hanson H, Cook HA, Nivin B. Fatalities associated with the 2009 H1N1 influenza A virus in New York city. Clinical Infectious Diseases. 2010 Jun 1;50(11):1498-504.
70. Shieh WJ, Blau DM, Denison AM, DeLeon-Carnes M, Adem P, Bhatnagar J, Sumner J, Liu L, Patel M, Batten B, Greer P. 2009 pandemic influenza A (H1N1): pathology and pathogenesis of 100 fatal cases in the United States. The American journal of pathology. 2010 Jul 1;177(1): 166-75.
71. Nakajima N, Sato Y, Katano H, Hasegawa H, Kumasaka T, Hata S, Tanaka S, Amano T, Kasai T, Chong JM, Iiduka T. Histopathological and immunohistochemical findings of 20 autopsy cases with 2009 H1N1 virus infection. Modern Pathology. 2012 Jan;25(1):1-3.
72. Perez-Padilla R, De La Rosa-Zamboni D, Ponce de Leon S, Hernandez M, Quiñones-Falconi F, Bautista E, Ramirez-Venegas A, Rojas-Serrano J, Ormsby CE, Corrales A, Higuera A. Pneumonia and respiratory failure from swine-origin influenza A (H1N1) in Mexico. New England journal of medicine. 2009 Aug 13;361(7):680-9.
